# Sale and Purchase of a Medical Practice

**Published:** 1899-11-11

**Authors:** 


### SALE AND PURCHASE OF A MEDICAL PRACTICE.

(*Continued from page* 84.)

Practice by an assistant is equivalent to practice by his principal, for *Qui facit per alium facit per se.* It would probably, in the absence of any express authority upon the point, be restrained by injunction. The establishment of a place of business such as a surgery is also unlawful. In Newington v. Bloxham (*Brit. Med. Jour.*, Feb. 6th, 1897) the defendant, who had been an assistant of the plaintiff, agreed not to practise within a five-mile radius after the termination, whether by notice or otherwise, of his period of service. He established a surgery within this radius. The Lord Chief Justice granted an injunction in the terms as asked. We have seen that to visit patients, even at their earnest solicitation, is a breach of contract. It would be otherwise if the patients were to travel to see the vendor in his new district, or probably if they saw him anywhere in consultation with and by the consent of the incoming practitioner.

It must not be supposed, however, that every interference with a purchaser's practice constitutes a breach of covenant.

The following case will serve to show how the Court is likely to interpret the covenant where the vendor has interfered at the request of the purchaser. In Rawlinson v. Clarke (11 M. and W. 187) the defendant assigned to the plaintiff his business as a surgeon and apothecary, covenanting that he should not nor would, directly or indirectly, by himself or in copartnership with any other person, carry on or exercise his practice or profession of surgeon or apothecary, or either of them, either by residing or visiting any patient within the distance of three miles from the then place of business of the defendant in Park Street. In case of any breach he undertook to pay £500 as and for liquidated damages, and not as a penalty. After the execution of this deed he attended several ladies in their confinements within three miles, and from one of them he received £14 14s. It was proved, however, that he attended these persons with the plaintiff's knowledge and consent, and in consequence of a request on his part that he should continue to visit the patients in order to keep the connection together. It was held that this did not constitute a breach of covenant.

It should, perhaps, be mentioned that even where a contract of service as an assistant may be put an end to by either party, upon giving a month's notice, the covenant not to practise within the prohibited radius may still be enforced. (Giles v. Hart, 1 L. T., 154.)

An important question sometimes arises as to whether the contract is personal; that is to say, whether the rights and liabilities connected with it pass to an assignee.

In Jacoby v. Whitmore (49 L. T., 335) a shopman had covenanted not to compete with his employer for a certain period. The employer sold the good-will to a third person, who retained the shopman in his service. It was held that the right to enforce the obligation passed with the good-will. From this we may infer that if a qualified assistant enters into a contract not to practise, the subsequent sale of his master's practice does not release him from his obligation.

Where two men in partnership sell their good-will, on pain of a penalty for infringement, a breach by one partner involves payment of the whole penalty. (Mercer v. Irving, El. Bl. and El., 563.)

Although the main object of this article is to discuss the legal as apart from the medico-ethical aspect of these contracts, a few instances of what has and not been ethically considered a breach of contract may be appended. Thus the acceptance of an invitation to lecture upon a topic of medical interest within the prescribed area is not a breach of contract, either legally or morally. And in the absence of any agreement or bond of any kind it is considered a breach of etiquette for a *quondam* assistant to hold himself out for practice in the district frequented by his former principal, although, of course, there is no legal remedy available against him. Further, although there is no implied legal undertaking on the part of a *locum tenens* not to practise in the immediate neighbourhood after his employment comes to an end, his doing so immediately is not considered honourable; but it is otherwise if he waits for a period of two or three years.

Having considered the various acts and defaults which are likely to constitute a breach of the contract, the consequences of such a breach remain to be considered. For this purpose it is necessary to refer once more to the common form used in these cases. It usually runs: That in case of any breach of the foregoing covenant "he will forthwith pay to the plaintiff £2,000, not in the nature of a penalty, but as liquidated damages."

Before citing any cases where the whole penalty has been enforced, it is necessary to allude to the policy of the law with regard to penalties. In all cases an action for damages lies for breach of contract, provided the contract is legal and in proper form. Sometimes, however, for the purpose of ensuring the due performance of an agreement the parties fix the damages beforehand, and quote the figure as a penalty in the body of the contract.

Nevertheless, a Court of Justice will occasionally go behind the express covenant and say: "Nay, it is clear that the plaintiff has not suffered damage to this extent; the enforcement of this penalty would be unjust, as the exact injury suffered is capable of easy estimation." This rule is applied, although the parties have agreed to regard the sum as liquidated, *i.e.*, as ascertained damages. But where the proper measure of damages is difficult, if not impossible, to ascertain, the Court will, as a rule, enforce the covenant according to its very letter. And so in the case of a contract for the sale of a medical practice. According to what scale, it is asked, could the value of a thing so shadowy be estimated? Bearing this in mind, the vendor must be prepared to pay the entire sum agreed upon, if he is found to be at fault, unless, indeed, he is willing to consent to an injunction granted by the Court at the instance of the purchaser with whom he has interfered.

The recovery and payment of a liquidated amount prevents an injunction being obtained, the liquidated sum being regarded as the value of the practice for the stated period. (Carnes v. Nesbit, 7 H. and N., 778.)

We have only alluded to the form of an ordinary bond for the purpose of showing the general nature of the restriction clause. Other terms are sometimes added. Thus, in the case of Reynolds v. Bridge (6 El. and Bl. 528), in addition to the ordinary covenant it was provided "that notwithstanding the clause lastly hereinbefore contained, the defendant may after the determination of the term act in consultation with other medical gentlemen in W—— or elsewhere; the defendant may also, if he think fit, after the determination of the term, attend midwifery cases in W—— or elsewhere within 20 miles thereof, but he shall pay one-half of the fees which he shall receive for each of such midwifery cases unto the plaintiff so long as he shall continue to practise as W——." It was here decided that the non-payment of the midwifery fees would not be within the covenant, and failure to pay them could not involve the defendant in liability to pay the penalty.

It is fortunate for the medical profession that disputes with regard to the interpretation of these covenants are few and far between. Indeed, the cases above referred to well nigh exhaust the list which can be made out after a search through the law reports. In conclusion, it may be well to remind the intending purchaser of a goodwill that any breach, however slight, on the part of the vendor gives him the right to claim the penalty, while a false and fraudulent representation as to the value of the practice, if its falsity can be clearly proved, entitles him to demand rescission of the contract.

